# Transcriptomic and volatile signatures associated with maize defense against corn leaf aphid

**DOI:** 10.1186/s12870-021-02910-0

**Published:** 2021-03-16

**Authors:** Lise Pingault, Suresh Varsani, Nathan Palmer, Swayamjit Ray, W. Paul Williams, Dawn S. Luthe, Jared G. Ali, Gautam Sarath, Joe Louis

**Affiliations:** 1 Department of Entomology, University of Nebraska-Lincoln, Lincoln, NE 68583 USA; 2 Wheat, Sorghum, and Forage Research Unit, USDA-ARS, Lincoln, NE 68583 USA; 3 Department of Entomology, Pennsylvania State University, University Park, PA 16802 USA; 4 Corn Host Plant Resistance Research Unit, USDA-ARS, Mississippi State, MS 39762 USA; 5 Department of Plant Science, Pennsylvania State University, University Park, PA 16802 USA; 6 Department of Biochemistry, University of Nebraska-Lincoln, Lincoln, NE 68583 USA

**Keywords:** Corn leaf aphid, Maize, Phytohormones, RNA-seq, Volatile organic compounds (VOCs)

## Abstract

**Background:**

Maize (*Zea mays* L.) is a major cereal crop, with the United States accounting for over 40% of the worldwide production. Corn leaf aphid [CLA; *Rhopalosiphum maidis* (Fitch)] is an economically important pest of maize and several other monocot crops. In addition to feeding damage, CLA acts as a vector for viruses that cause devastating diseases in maize. We have shown previously that the maize inbred line Mp708, which was developed by classical plant breeding, provides heightened resistance to CLA. However, the transcriptomic variation conferring CLA resistance to Mp708 has not been investigated.

**Results:**

In this study, we contrasted the defense responses of the resistant Mp708 genotype to those of the susceptible Tx601 genotype at the transcriptomic (mRNA-seq) and volatile blend levels. Our results suggest that there was a greater transcriptomic remodeling in Mp708 plants in response to CLA infestation compared to the Tx601 plants. These transcriptomic signatures indicated an activation of hormonal pathways, and regulation of sesquiterpenes and terpenoid synthases in a constitutive and inducible manner. Transcriptomic analysis also revealed that the resistant Mp708 genotype possessed distinct regulation of ethylene and jasmonic acid pathways before and after aphid infestation. Finally, our results also highlight the significance of constitutive production of volatile organic compounds (VOCs) in Mp708 and Tx601 plants that may contribute to maize direct and/or indirect defense responses.

**Conclusions:**

This study provided further insights to understand the role of defense signaling networks in Mp708’s resistance to CLA.

**Supplementary Information:**

The online version contains supplementary material available at 10.1186/s12870-021-02910-0.

## Background

Maize (*Zea mays* L.) is one of the most significant cereal crops grown in the world. Moreover, maize is an important staple in many countries and provides at least 30% of the food calories for more than 4.5 billion of people in many developing countries [1]. Consequently, any stress that negatively impacts maize production, for example, plant diseases and pest outbreaks are dangerous and could lead to a cascading effect on food security. Maize is attacked by a plethora of insect pests that feed both above and belowground [2].

Aphids are sap-sucking insect pests that significantly impact crop yield loss [3, 4]. Indirectly, aphids also cause damage to the plants by vectoring plant viral diseases [5]. Corn leaf aphid [CLA; *Rhopolosiphum maidis* (Fitch)] is the most commonly found sap-sucking insect pest on maize [2, 6]. Aphids, including CLA, feeds from the vascular tissues using stylets present in their mouthparts [3, 7, 8]. Apart from causing direct yield loss, CLA feeding acts as a vector for viruses such as maize dwarf mosaic virus and maize leaf fleck virus [9, 10]. Additionally, CLA feeding covers maize plants with honeydew (the digestive waste product of aphids), that leaves a sticky deposit on the plants and causes mold, thereby disrupting and/or reducing photosynthetic efficiency [11].

We have previously shown that the maize inbred line Mp708, which was developed by classical plant breeding from a cross between the insect resistant Mp704 and susceptible Tx601 plants, provides heightened resistance to CLA [12–15]. Feeding by CLA triggers the accumulation of *mir1* transcripts, which encodes Maize insect resistance1-Cysteine Protease (Mir1-CP) defensive protein [13, 14]. Furthermore, foliar feeding by CLA rapidly triggers distal belowground accumulation of *mir1* through an unknown mechanism [13, 14]. The transport of defense-related compounds from roots to shoots in response to foliar insect infestation has been documented in other systems [15, 16]. For example, green peach aphid (*Myzus persicae*) feeding on *Arabidopsis thaliana* foliage induced the expression of *LIPOXYGENASE 5* (*LOX5*) transcript in roots [16]. *LOX5*, which encodes a 9-LOX, and/or a LOX5-dependent product(s) synthesized in the roots were likely translocated to the shoots through the vascular system, and enhanced aphid colonization [16, 17]. It has also been shown that Mir1-CP accumulation in the roots after foliar feeding by CLA provided enhanced resistance to root-feeding herbivores [14]. In addition, we have demonstrated that constitutively elevated levels of 12-oxo-phytodienoic acid (OPDA), an intermediate in the jasmonic acid (JA) biosynthesis pathway, contributed to enhanced callose accumulation and resistance to CLA in Mp708 plants [18, 19]. Genetic and pharmacological analyses, however, pointed that the OPDA-mediated resistance to CLA is independent of the JA pathway [18]. Furthermore, OPDA also regulated the expression of ethylene (ET) biosynthesis and receptor genes, which act as a significant component in regulating *mir1* expression in providing resistance to CLA [13, 18].

Besides direct defenses (for example, Mir1-CP-mediated defenses in maize), plants also activate indirect defenses, which include various plant volatiles that involve a tritrophic interaction between the plant, the insect, and a predatory insect [20]. Plant volatile organic compounds (VOCs) are mainly comprised of terpenoids, fatty acid derivatives, phenylpropanoids, and benzenoids [21]. These indirect defenses can be constitutive or inducible in nature. For instance, constitutive or herbivore-induced methyl salicylate (MeSA) attracts predatory insects to their host plants, which helps to curtail the herbivorous prey population [22, 23]. For example, feeding by soybean aphids (*Aphis glycines*) on soybeans (*Glycine max*) induced MeSA that attracted predatory beetles, *Coccinella septempunctata*, to the host plants, thereby limiting aphid proliferation [23]. In addition, some of these VOCs act as airborne signals that potentially activate defense responses to subsequent insect herbivory in undamaged regions within the plant or to adjacent plants [24–26].

In this study, we coupled transcriptomic and volatile profiling to unveil the modulation of early defense mechanisms in leaves and roots of Tx601 and Mp708 plants before and after CLA infestation. Our results suggest that the resistant Mp708 genotype uniquely activates different pathways involved in plant defense mechanisms before and after aphid infestation that may contribute to resistance to CLA, whereas the modulation of defense pathways before or after aphid infestation were not accompanied by enhanced defense against CLA in the susceptible Tx601 genotype. Finally, volatile profiling of aphid uninfested plants indicated that the CLA resistant Mp708 genotype emitted constitutive low levels of VOCs compared to the susceptible Tx601 genotype. The VOC data coupled with the transcriptomic changes suggest that the Mp708 genotype may be better adapted to activate defenses in response to CLA feeding.

## Results

### Maize transcriptomic responses to CLA infestation

**Fig. 1:**
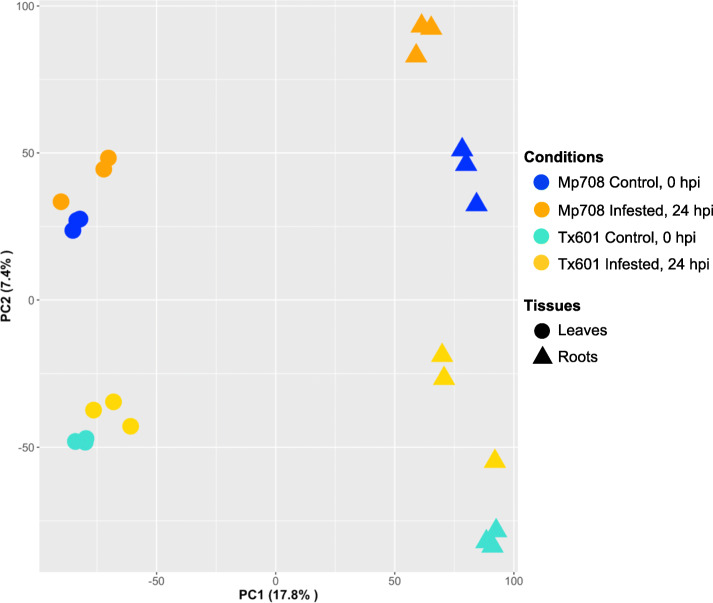
Principal component analysis **(**PCA) of all the 38,897 genes expressed in at least one condition. Conditions are represented with colors (Mp708 control = blue, Tx601 control = turquoise, Mp708 infested = orange, Tx601 infested = yellow) and the tissues with the shape (leaves = circle and roots = triangle)

RNA-seq was used to identify transcriptomic changes in response to aphid feeding on both susceptible and resistant maize genotypes. Read mapping was performed on the maize reference genome (v4; www.phytozome.org), which indicated that 38,897 genes were expressed in at least one of the eight conditions. A principal component analysis (PCA) of the 38,897 genes was performed and PC1 accounted for 17.8% of the variance, separating the transcriptomes by tissue, and PC2 accounted for 7.4% of the variance, separating the transcriptomes by genotype and treatment (Fig. 1). Differential expression was investigated in two ways: (i) for each genotype between the two time points (0 and 24 h post infestation, hpi), and (ii) for each time point between both genotypes. Differentially expressed genes (DEGs) were defined with a significant expressed difference (*P* < 0.05) and at least a two-fold change relative to the respective control: 24 hpi vs. 0 hpi, or genotype: Mp708 vs. Tx601. In total, 25,180 unique genes were differentially expressed. Among these, 3964 and 8074 genes were differentially expressed after CLA infestation in Mp708 leaves and roots, respectively, and 3463 and 7305 were differentially expressed after CLA infestation in Tx601 leaves and roots, respectively. The repartition of the DEGs for each comparison has been summarized in the Table 1.

**Table 1:** Number of differentially expressed genes (DEGs) for each comparison

Treatment 1	Treatment 2	Upregulated (Treatment 2 / Treatment 1)	Downregulated (Treatment 2 / Treatment 1)	Total DEGs
Mp708 (0 hpi) Leaves	Mp708 (24 hpi) Leaves	2226 (56.1%)	1738 (43.8%)	3964
Tx601 (0 hpi) Leaves	Tx601 (24 hpi) Leaves	1578 (45.6%)	1885 (54.4%)	3463
Mp708 (0 hpi) Roots	Mp708 (24 hpi) Roots	3305 (40.9%)	4769 (59.1%)	8074
Tx601 (0 hpi) Roots	Tx601 (24 hpi) Roots	2972 (40.6%)	4333 (59.4%)	7305
Mp708 (0 hpi) Leaves	Tx601 (0 hpi) Leaves	3218 (54.7%)	2662 (45.3%)	5880
Mp708 (24 hpi) Leaves	Tx601 (24 hpi) Leaves	3045 (48.5%)	3230 (51.6%)	6275
Mp708 (0 hpi) Roots	Tx601 (0 hpi) Roots	3073 (52.6%)	2773 (47.4%)	5846
Mp708 (24 hpi) Roots	Tx601 (24 hpi) Roots	3720 (55.6%)	2969 (44.4%)	6689

*hpi* hours post infestation; Percentage of DEGs up or downregulated are indicated in parenthesis

**Fig. 2:**
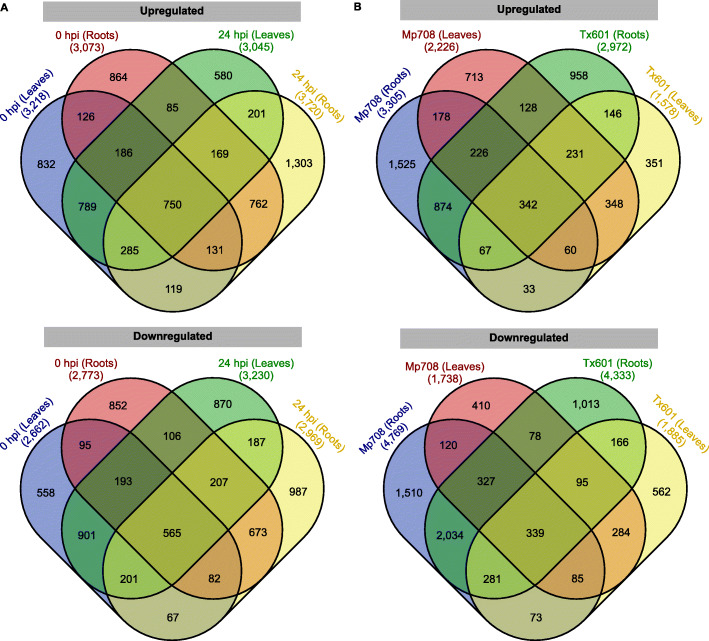
Venn diagrams of differentially expressed genes (DEGs) in Mp708 and Tx601 genotypes. The number of DEGs are indicated in parenthesis. **a** Number of up or downregulated genes in Mp708 compared to Tx601 genotypes for each tissue at 0 h post infestation (hpi) or 24 hpi and **b** Number of up or downregulated genes at 24 hpi for each tissue and genotype

After 24 h of aphid feeding, thousands of genes varied in their expression level. Among the 25,180 DEGs, 1509 and 981 were commonly upregulated in the roots and leaves, respectively (Fig. 2a). On the other hand, 2981 and 803 were commonly downregulated in root and leaves, respectively (Fig. 2a). The proportion of DEGs up or downregulated after infestation was impacted by the maize genotype (Table 1). In uninfested plants, 3218 (54.7%) of the 5880 DEGs were upregulated in Mp708 leaves, whereas 3045 (48.5%) of the 6275 DEGs were upregulated in Mp708 leaves 24 h after CLA infestation (Table 1). For the root tissues, 3073 (52.5%) and 3720 (55.6%) genes were upregulated in the Tx601 uninfested and infested roots, respectively (Table 1). In addition, the number of genes up or downregulated in each tissue for each genotype were also monitored. Eight hundred and six genes were upregulated in Mp708 leaves and roots, whereas, 876 genes were downregulated in the Mp708 leaves and roots (Fig. 2b).

**Fig. 3:**
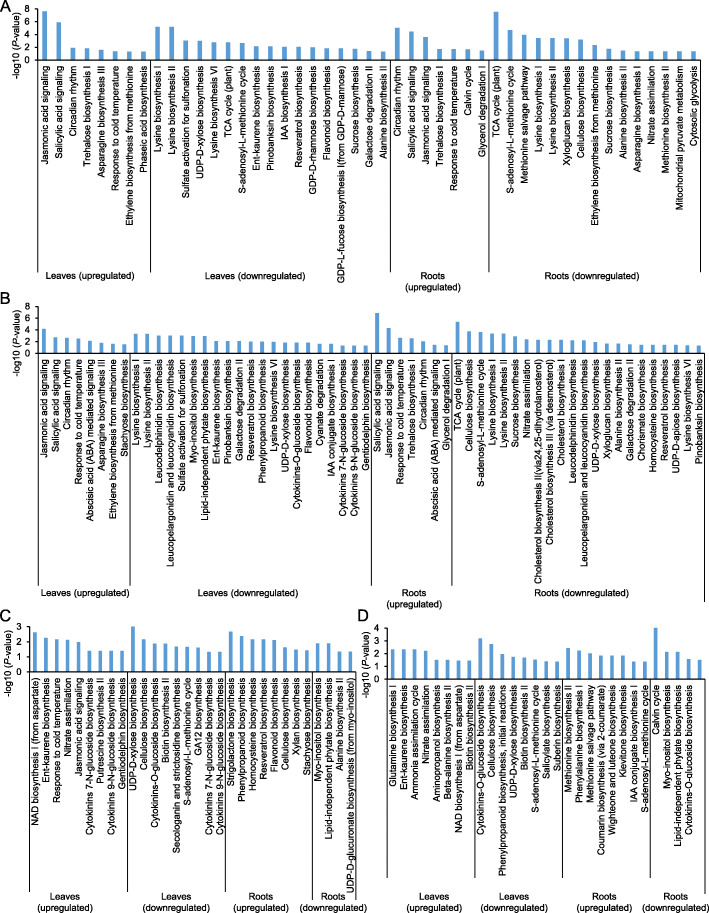
Comparison of pathway enrichment analysis in leaves and roots of Mp708 and Tx601 genotypes. **a** Mp708 differentially expressed genes (DEGs) up or downregulated at 24 h post infestation (hpi), **b** Tx601 DEGs up or downregulated at 24 hpi, **c** DEGs up or downregulated in Mp708 compared to Tx601 at 0 hpi, and **d** DEGs up or downregulated in Mp708 compared to Tx601 at 24 hpi. Barplot with significant *P*-values (< 0.05) were sorted using the descending negative logarithmic adjusted *P*-value of the enrichment analysis for each comparison

To further understand the role of different plant defense pathways involved in the response to CLA infestation, pathway enrichment was investigated in the up or downregulated genes. After 24 h of CLA infestation, genes upregulated in leaves were related to JA signaling, SA signaling or ethylene biosynthesis from methionine in both genotypes (Fig. 3a and b). However, genes with function related to abscisic acid (ABA)-mediated signaling were enriched only in Tx601 genotype (Fig. 3b). Similar observations were made for the belowground tissues: SA signaling, JA signaling, response to cold temperature were part of the functions upregulated after CLA infestation in both genotypes (Fig. 3a and b). Also, ABA-mediated signaling function was found enriched only in the Tx601 roots after CLA infestation (Fig. 3b). “Flavonoid biosynthesis” or “Lysine biosynthesis” were part of the pathways downregulated in the leaves of both genotypes (Fig. 3a and b). However, “sucrose biosynthesis” or auxin biosynthesis “IAA biosynthesis I” pathways were downregulated in Mp708 leaves (Fig. 3a) and cytokinin biosynthesis pathway (“cytokinin 7-N-glucoside biosynthesis”, “cytokinin 9-N-glucoside biosynthesis” or cytokinin-O-glucoside biosynthesis) or “phenylpropanoid biosynthesis” were part of the pathways downregulated in Tx601 leaves only (Fig. 3b). Common downregulated pathways in the roots were related to “Lysine biosynthesis I and II”, “TCA cycle”, “cellulose biosynthesis” or “nitrate assimilation”. Interestingly, “Methionine salvage pathway”, “ethylene biosynthesis from methionine” pathways were downregulated in Mp708 roots. Downregulated pathways specific to Tx601 roots were related to cholesterol biosynthesis (“cholesterol biosynthesis I”, “cholesterol biosynthesis II”, “cholesterol biosynthesis III”) or UDP-D-xylose biosynthesis (Fig. 3b).

By comparing both data from uninfested plants of both genotypes, genes downregulated in Mp708 leaves were associated with “cellulose biosynthesis”, “cytokinin biosynthesis” or “biotin biosynthesis”, while upregulated genes were associated with functions such as “JA signaling” and “nitrate assimilation” (Fig. 3c). Genes downregulated in Mp708 compared to Tx601 leaves at 24 hpi were involved in cytokinin biosynthesis pathways (cytokinin-O-glucoside biosynthesis) or cellulose biosynthesis. Interestingly, upregulated genes in Mp708 compared to Tx601 leaves at 24 hpi were enriched in nitrate assimilation (Fig. 3d).

The comparison of the root transcriptomes between the two genotypes indicated that downregulated genes in roots from Mp708 plants were associated with functions enriched in “Myo-inositol biosynthesis”, “Alanine biosynthesis II” or “Lipid-independent phytate biosynthesis” while upregulated genes had functions enriched in “Strigolactone biosynthesis”, “phenylpropanoid biosynthesis” or “flavonoid biosynthesis”. After CLA infestation, downregulated genes in Mp708 roots had functions related to “Calvin cycle”, “cytokinin-O-glucoside biosynthesis” while upregulated genes had functions related to “methionine biosynthesis II”, “phenylalanine biosynthesis I” or “methionine salvage pathway” (Fig. 3c and d).

### Gene co-expression clusters were specific either to genotype or tissue

**Fig. 4:**
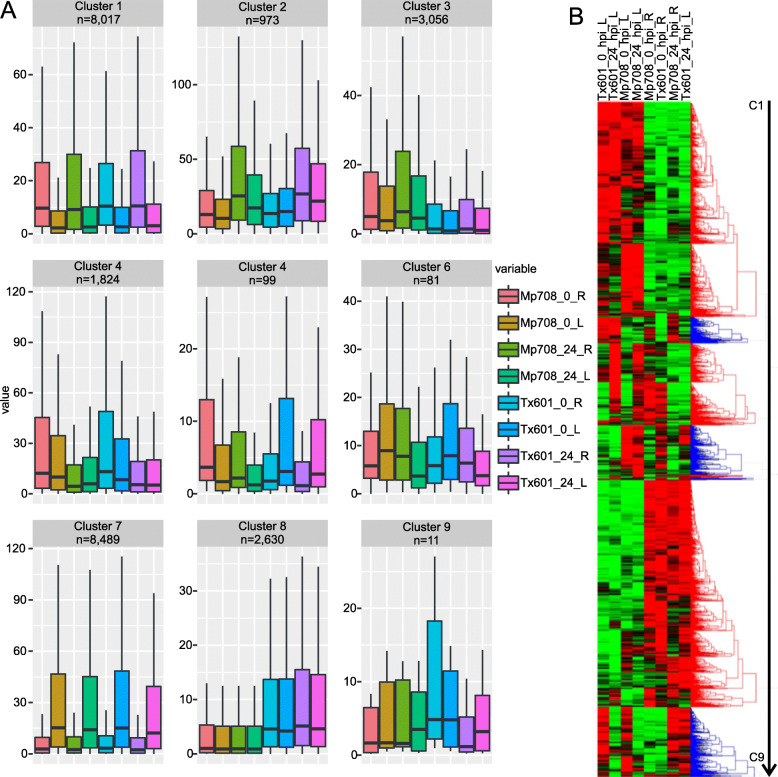
Co-expression clusters of the 25,180 differentially expressed genes (DEGs). **a** Expression patterns of genes assigned to nine clusters, ‘n’ indicates the number of DEGs for each module. **b** Hierarchical clustering of the DEGs. Red indicates upregulated genes and green indicates downregulated genes. The different clusters are represented in blue or red. L = leaves; R = roots; hpi = hours post infestation

A hierarchical clustering analysis was performed on the 25,180 non-redundant DEGs with a minimal similarity to establish the clusters set to 0.834, which is the Pearson correlation significant at the *P*-value threshold of 0.01, and nine co-expression clusters were found: C1 (Cluster 1) to C9 (Fig. 4). The number of genes in each cluster varied from 11 (C9) to 8489 (C7) (Fig. 4). Two clusters were composed of genes expressed specifically in uninfested and infested roots or leaves: C1 (8017 genes; genes expressed in root tissues) and C7 (8489; genes expressed in leaf tissues). Two clusters were composed of genes upregulated specifically in Mp708 roots or Tx601 roots and leaves: C3 (3056 genes upregulated in Mp708) and C8 (2630 genes upregulated in Tx601). C4 (1,824) were composed of genes upregulated in uninfested conditions for both genotypes. The three remaining clusters (C5, 99 genes; C6, 81 genes; and C9, 11 genes) were composed of genes with a transient expression pattern in all the conditions (Fig. 4).

### The activation of the defense mechanisms was specific to each genotype

We identified 3056 genes expressed specifically in the root tissues of the Mp708 genotype (C3, Fig. 4). *mir1* (*Zm00001d036542*) was found part of the C3 and was upregulated in the roots of foliar infested CLA plants (Supplemental Table 1). The functions of the genes that were part of C3 were annotated as “metalothionein 2B” (*Zm00001d048611*), “low temperature and salt responsive protein” (*Zm00001d024778*), “Dormancy/auxin associated family protein” (*Zm00001d032422*), and “Methylenetetrahydrofolate reductase family protein” (*Zm00001d029853*). In addition, a member of the PLAC8 family protein (*Zm00001d039776*) was found part of the C3 and was upregulated in Mp708 roots obtained from CLA-infested plants. Gene ontology enrichment analysis showed that co-expressed genes in C3 were enriched in “transferase complex”, “phosphatidylinositol 3-kinase complex”, “PAS complex”, “vacuolar membrane” or “vacuole” (cellular component) (Supplemental Table 2). The KEGG pathway analysis revealed genes enriched in JA biosynthesis and signaling pathway, “Ethylene mediated signaling” or “Thiamine biosynthesis” (Supplemental Table 2).

Among the 2630 genes specifically upregulated in Tx601 plants (C8), the genes with the higher cumulative expression levels were annotated as “PHE ammonia lyase 1” (*Zm00001d017274*), “B12D protein” (*Zm00001d037275*), “Low temperature and salt responsive protein family” (*Zm00001d008200*) or “calmodulin-like 11” (*Zm00001d005895*). The KEGG enrichment analysis revealed functions as “beta-alanine biosynthesis II”, “NAD biosynthesis I”, “Pantothenate biosynthesis I/II” or “Strigolactone signaling” (Supplemental Table 2).

### Repartition of the transcription factors was not equal in the different expression clusters

**Fig. 5:**
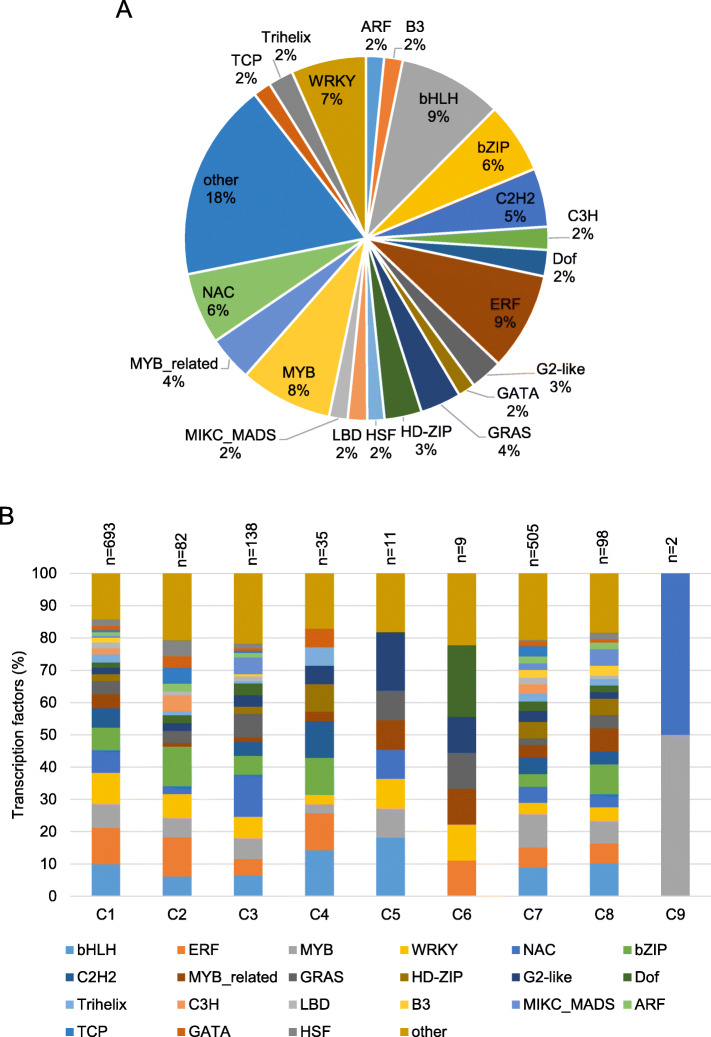
Proportion of transcription factors (TFs). **a** Repartition of the TF families among the differentially expressed genes (DEGs) and, **b** Proportion of TFs per cluster. ‘*n*’ indicates the number of TF for each module

Transcription factors (TFs) play a crucial role in regulating plant responses to insect herbivory and different classes of TFs can differentially modulate these responses [27]. In total, 1573 TFs were found differentially expressed in our dataset (Supplemental Table 3). The majority of the TFs were part of the C1 (693 TFs; 44%) and C7 (505 TFs; 32.1%) (Supplemental Table 3). Among the TFs, the families most represented were bHLH (145 TFs), ERF (137 TFs), MYB (129 TFs) and WRKY (105 TFs) (Fig. 5a). Further repartition of the TFs in C3 (138 TFs), NAC (18 TFs) represent the higher proportion of the TFs, followed by GRAS (10 TFs), bHLH (9 TFs), MYB (9 TFs) and WRKY (9 TFs) (Fig. 5b). In the C8, 98 of the DEGs were TFs, including 10 bHLH, 9 bZIP, 7 MYB and 7 MYB-related (Fig. 5b; Supplemental Table 3).

### Genes involved in phytohormone biosynthesis were modulated by aphid feeding

**Fig. 6:**
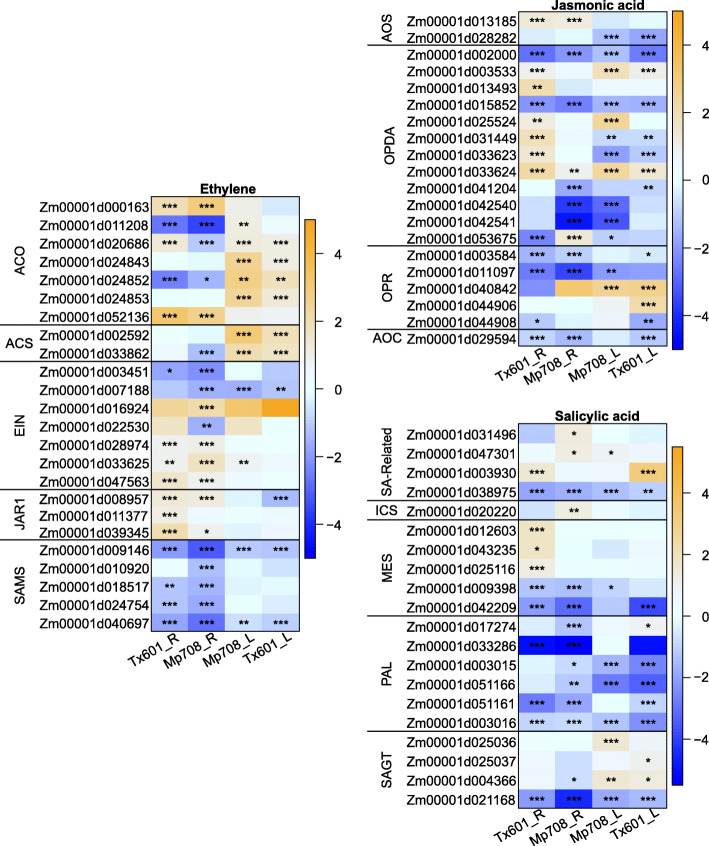
Heatmap of the expression CLA feeding-induced fold-change of each gene involved in different hormone pathways. Each column corresponds to a condition for each genotype (R: root or L: leaves). Each cell contains the corresponding log_2_(fold-change 24 hpi/0 hpi) level (blue-orange scale) and adjusted *P*-value (*** < 0.001, 0.001 < ** < 0.01, 0.01 < * < 0.05)

To identify signatures of hormonal responses in infested plants, we filtered the genes based on their function in hormonal pathways (Fig. 6; Supplemental Table 4).

#### Jasmonic acid

JA biosynthesis starts with α-linolenic acid (18:3), which is the substrate for lipoxygenase (LOX) enzymes. The *LOX* gene family in maize contains 13 LOX encoding loci (*ZmLOX1–13*) [28]. Further, LOX enzymes were subdivided into two groups depending on where they oxygenate α-linolenic acid, 9-lipoxygenases and 13-lipoxygenases. There are seven 9-lipoxygenases (*ZmLOX1, 2, 3, 4, 5, 6, 12*) and six 13-lipoxygenases (*ZmLOX7, 8, 9, 10, 11, 13*) in maize. 13-lipoxygenases are the first step to the production of JA, while products of 9-lipoxygenases can still have defensive functions against insect herbivory [29].

Among the 13 *LOX* annotated genes, seven were differentially expressed and encoded 9-lipoxygenases: *Zm00001d033623* (*ZmLOX3*), *Zm00001d042540* (*ZmLOX2*) and *Zm00001d042541* (*ZmLOX1*), *Zm00001d033624* (*ZmLOX4*), *Zm00001d025524* (*ZmLOX7*), *Zm00001d003533* (*ZmLOX8*), *Zm00001d002000* (*ZmLOX6*). Among these genes, three were upregulated in Mp708 leaves (*ZmLOX4*, *ZmLOX7* and *ZmLOX8*) and two were upregulated in Tx601 leaves (*ZmLOX4* and *ZmLOX8*) (Supplemental Table 4). *ZmLOX6* encoding a “PLAT/LH2 domain-containing lipoxygenase family protein” was downregulated in roots and leaves of both genotypes. Two were downregulated in Mp708 roots and leaves only (*ZmLOX1* and *ZmLOX2*). One was upregulated in leaves of both cultivars (*ZmLOX8*). Among the 13-lipoxygenases, one locus *Zm00001d053675* (*ZmLOX10*) was up and downregulated in both Mp708 and Tx601 roots, respectively. *Zm00001d013493* (*ZmLOX5*) was induced in Tx601 roots only (Fig. 6; Supplemental Table 4).

Allene oxide synthase (AOS) catalyzes the next enzymatic step towards JA production [30]. There are six putative AOS encoding loci in maize, only two of which showed significant expression in our dataset. One locus (AOSa; *Zm00001d002592*) was induced by CLA in roots of both maize genotypes, one locus (AOSb; *Zm00001d028282*) had reduced expression in the leaves of both lines as a result of CLA infestation. Allene oxide cyclase (AOC) and oxophytodienoic acid reductase (OPR) catalyze two additional enzymatic reactions required for JA production [31]. Maize has two AOC encoding loci, one whose expression levels were unaffected by CLA while the second AOC encoding locus had reduced expression in all tissues and lines. Eight putative OPR encoding loci are found in the maize genome, five of which were impacted by CLA infestation. One locus (*OPRd*) showed no expression in roots but was induced in leaves of both genotypes. The expression of the second OPR locus (*OPRb*) was downregulated in the roots of both genotypes, but relatively unchanged in leaf tissues.

Another JA associated gene in Arabidopsis is *JAR1*, which conjugates JA to isoleucine and is induced by auxin [32]. Interestingly, *JAR1* has also been shown to conjugate JA to ACC (the intermediate in ethylene biosynthesis) [32]. Five putative JAR1 encoding loci have been reported in maize (*ZmJAR1a*,*b* and *ZmJAR2a-c*) [33], although only *ZmJAR1a*, *ZmJAR1b*, and *ZmJAR2a* had significant expression in our dataset. *ZmJAR1c* (*Zm00001d011377*) and *ZmJAR1d* (*Zm00001d039345*) were induced in Tx601 leaves and unchanged in both tissues in Mp708 and Tx601 roots; *ZmJAR1a* (*Zm00001d008957*) had reduced expression in Tx601 leaves, and was induced in Mp708 and Tx601 roots after CLA infestation (Fig. 6; Supplemental Table 4).

#### 12-oxo-phytodienoic acid (OPDA)

Among the eight OPDA annotated genes (*OPR*), five were differentially expressed: *OPR1* (*Zm00001d044908)*, *OPR2* (*Zm00001d044906*), *OPR4* (*Zm00001d011097*), *OPR5* (*Zm00001d003584*) and *OPR6* (*Zm00001d040842*). *OPR6* was upregulated in both genotypes leaves at 24 hpi. *OPR2* was upregulated at 24 hpi in Tx601 leaves, while *OPR1* and *OPR4* were downregulated at 24 hpi in Tx601 and Mp708 leaves. In addition, *OPR4* was downregulated in Mp708 and Tx601 roots and Mp708 leaves only (Fig. 6; Supplemental Table 4).

#### Ethylene

Ethylene biosynthesis starts with methionine, which is converted to S-adenosyl-L-methionine (SAM) by SAM-synthase (SAMS) [34]. Further, SAM is converted to 1-aminocyclopropane-1-carboxylic acid (ACC) by ACC synthase (ACS). ACS also produces MTA (methylthioadenosine), which is recycled back into SAM via the Yang cycle [34]. Ethylene is then produced from ACC by ACC oxidase (ACO). There are five maize loci encoding ACS enzymes (ACSa: *Zm00001d002592*, ACSb: *Zm00001d026060*, ACSc: *Zm00001d033862*, ACSd: *Zm00001d039487*, ACSe: *Zm00001d045479*), all of which were expressed our dataset. Only two genes (*ACSa* and *ACSc*) were differentially expressed, both induced at 24 hpi in the leaves of both genotypes and the expression of *ACSc* was reduced in Mp708 roots (Supplemental Table 4).

There are 13 putative ACO encoding loci in maize [35] and seven of them show significant expression variation in our dataset. Five and four of the *ACO* were upregulated in leaves of Mp708 and Tx601 plants, respectively, two were upregulated in the roots of both genotypes (*ACOa* and *ACOi*), and two were downregulated in both root genotypes (*ACOb* and *ACOf*). However, one gene (*ACOd*) was up and downregulated in Tx601 and Mp708 roots, respectively (Supplemental Table 4).

Another important portion of ethylene biosynthesis is the regeneration of the starting substrate, SAM, through the Yang cycle [34]. This cycle has six enzymatic steps, ending with the production of SAM by SAM synthase (SAMS) [34]. In our dataset, five loci were differentially expressed, and all had reduced expression primarily in the roots of both genotypes after CLA infestation (Supplemental Table 4). Similarly, EIN2 in Arabidopsis is required for ethylene signaling [36]. One copy of *ZmEIN2* (*Zm00001d013492*) showed increased expression in Tx601 compared to Mp708 at both 0 and 24 hpi in leaves and roots. Similarly, another member of the *EIN* gene family (*Zm00001d013492*) showed the same expression pattern. EIN3 and EIL1 are essential TFs for ethylene signaling in Arabidopsis and ERF1 is an immediate target of EIN3 in Arabidopsis [37]. ERF1 is part of the ERF (Ethylene response factor), which is a large class of AP2/EFR domain-containing TF. Among the annotated ERF/AP2 TF, 13 were differentially expressed between the two time points. Three were exclusively upregulated at 24 hpi in Mp708 (*Zm00001d006169*, *Zm00001d006170* and *Zm00001d003884*) while nine were upregulated in both genotypes and tissues (*Zm00001d017592*, *Zm00001d002618*, *Zm00001d036003*, *Zm00001d002025*, *Zm00001d021207*, *Zm00001d021208*, *Zm00001d002762*, *Zm00001d031673*, *Zm00001d025281*). Only one gene (*Zm00001d016262*) was downregulated at 24 hpi in Mp708 leaves and Tx601 roots.

#### Salicylic acid

SA biosynthesis has been linked to two different pathways: the isochorismate (IC) pathway and phenylalanine ammonia-lyase (PAL) pathway [38, 39]. Among the genes involved in the SA pathway, 19 were found differentially expressed in our data set (Supplemental Table 4). Only one gene (*ICS2*, *Zm00001d020220*) of the IC pathway was differentially expressed in our data set and was upregulated in Mp708 roots. Genes encoding “PHE ammonia lyase 1/2” were downregulated after aphid attack in Mp708 roots (*Zm00001d051161*, *Zm00001d033286*, *Zm00001d051166*, *Zm00001d017274*), Tx601 roots (*Zm00001d033286*, *Zm00001d051161*), Mp708 and Tx601 leaves (*Zm00001d051166*, *Zm00001d003015*, *Zm00001d003016*). Among the five DEGs encoding “methyl esterase”, three were upregulated only in Tx601 roots and two were downregulated in the Mp708 roots and Tx601 roots and leaves. Four DEGs encoding “UDP-glycosyltransferase” potentially linked to glycosylation of SA were upregulated in leaves of both genotypes after CLA infestation, while one was downregulated in all the conditions (Fig. 6; Supplemental Table 4).

### Aphid uninfested susceptible maize genotype has constitutively elevated levels of terpenes

Terpenoids are synthesized by several enzymes including terpene synthase (TPS) and are an integral part of plant interactions with the environment [40]. In our dataset, 11 *TPS* were differentially expressed after aphid infestation: *TPS1*, *TPS2*, *TPS3*, *TPS5*, *TPS7*, *TPS8*, *TPS10*, *TPS11*, *TPS17*, *TPS23* and *TPS26* (Supplemental Table 5). *TPS5* and *TPS23* were upregulated in CLA uninfested leaves of Mp708 compared to the other *TPS* genes that were upregulated in CLA uninfested Tx601 leaves.

To identify the variation in VOCs and terpenoids between the susceptible (Tx601) and resistant (Mp708) maize genotypes before CLA infestation, emitted plant volatiles were collected in a push-pull system for 8 h and analyzed using GC-MS. Table 2 summarizes the results of the 15 VOCs that were identified by the GC-MS. Eight VOCs were differentially emitted in both genotypes and were released in higher abundance in the Tx601 genotype, but six VOCs were absent for the Mp708 genotype. Among the six VOCs that were not present in the Mp708 genotype, five were volatile sesquiterpenes (α-ylangene, germacrene D, (E)-β-farnesene, α-muurolene and δ-cadinene), and one aromatic compound 3-hexen-1-ol-acetate. Two compounds linalool (monoterpene) and (E)-4,8-dimethylnona-1,3,7-triene (or DMNT; sesquiterpene) were present in both genotypes but significantly enriched in Tx601 plants (Table 2; Supplemental Fig. 1).

**Table 2:** Constitutive volatile organic compounds (ng of compound/g of tissue) emitted by Mp708 and Tx601 genotypes

Compound	Mp708	Tx601	***P***-value
Cis-1-hexenol	571.95 ± 132.41	600.34 ± 131.73	0.84
Nonane	697.66 ± 366.98	388.96 ± 200.11	0.762
Citronellene	411.71 ± 189.93	242.89 ± 148.68	0.369
3-Hexen-1-ol acetate	0	593.54 ± 158.48	< 0.001*
Decane	1219.20 ± 513.52	835.65 ± 442.80	0.645
Linalool	51.82 ± 37.81	278.60 ± 82.75	0.039*
Undecane	286.27 ± 126.71	195.59 ± 118.31	0.353
(E)-4,8-Dimethylnona-1,3,7-triene	134.57 ± 58.76	352.62 ± 82.48	0.023*
Indole	0	15.59 ± 10.21	0.149
Methyl salicylate	235.41 ± 56.21	159.59 ± 51.14	0.407
α-Ylangene	0	969.36 ± 125.88	< 0.001*
Germacrene D	0	110.73 ± 41.13	0.004*
(E)-β-farnesene	0	71.88 ± 21.68	0.004*
α-Muurolene	0	163.22 ± 36.45	< 0.001*
δ-Cadinene	0	218.08 ± 47.98	< 0.001*

## Discussion

Maize inbred line, Mp708, provides resistance to diverse feeding guilds of insect pests [13, 41, 42]. Results presented in this study and our previous studies [13, 14, 18] demonstrated that different pathways are required for activating defenses in Mp708 against different insect pests. For example, combined actions of ET and JA are required for providing Mir1-CP-mediated defense against chewing insects in Mp708, whereas CLA feeding-induced expression of *mir1* in Mp708 genotype is independent of the JA pathway and is dependent only on the ET pathway [13, 43, 44]. Furthermore, OPDA acts upstream of ET pathway in activating *mir1*-dependent defenses in maize against CLA [18]. In the current study, we unravelled that higher number of upregulated DEGs were found in Mp708 compared to Tx601 plants and a timely transcriptional reprogramming in Mp708 genotype after CLA infestation may be involved in Mp708’s resistance to CLA.

### Transcriptomic response specific to Mp708 genotype

Among the maize defense mechanisms toolbox, Mir1-CP has been characterized as a key defense protein in response to CLA attack [13, 14]. Foliar feeding by CLA induced *mir1* expression not only at the site of infestation but also distally in the roots, with ET playing a central role in the regulation of *mir1* [13]. This study further confirms that *mir1* was upregulated in Mp708 roots after CLA infestation on leaves and gene function enrichment analysis predicted a role for ET in the regulation of *mir1*. We also found that the *NACs* represent the highest proportion (18 genes) of TF families co-expressed with *mir1*. NACs are plant-specific TFs and several previous studies have shown that numerous NACs could be regulated by ET. For example, ANAC074 (AT4G28530) was reported to bind the promoter of ET responsive genes and stress responsive genes [45]. In tomato, at least one *NAC* gene was influenced by the mutation of the gene encoding an ET receptor [46]. Microarray analysis revealed that about one-third of *NAC* genes were regulated by the application of 1-aminocyclopropane-1-carboxylate, a direct precursor of ET [47]. In Arabidopsis, the mutation of *EIN2* (*ETHYLENE INSENTIVE 2*) blocked the induction of *NAC092*/*AtNAC2/ORESARA1* (*ORE1*) expression under salt conditions [48]. Here, two genes co-expressed with *mir1* (C3) encoded proteins annotated with functions related to low temperature/salt.

### Modulation of genes involved in phytohormonal pathways before and after CLA infestation

Phytohormones play a key role in modulating plant defense against sap-sucking aphids [3, 4, 8, 49]. Here, we investigated the transcriptomic response of the genes involved in phytohormone biosynthesis in response to CLA infestation in the resistant and susceptible genotypes. Genes related to JA biosynthesis were upregulated in aphid-uninfested leaves of the resistant Mp708 genotype compared to leaves from uninfested Tx601 genotype. This is consistent with our previous observation that Mp708 plants had constitutive elevated levels of JA prior to insect herbivory [13, 50]. After CLA infestation, JA-related plant defense mechanisms were activated in the leaves of the resistant Mp708 genotype. OPDA, an intermediate of JA biosynthesis can also act as a signaling compound on its own, and notably, OPDA has been shown to impact callose formation and contributed to enhanced resistance in Mp708 against CLA [18]. Although Mp708 plants had elevated levels of JA, genetic analysis, however, indicated that OPDA-mediated resistance to CLA was independent of the JA pathway [18]. Genes upregulated in the susceptible Tx601 genotype were also associated with plant defense mechanisms, however, these mechanisms did not prevent extensive damage arising from CLA infestation. Interestingly, we found that CLA-infested Tx601 leaves had higher expression of genes involved in the biosynthesis of a tetrasaccharide - stachyose. In plants, aphid feeding-induced trehalose, a non-reducing α,α-1,1-linked glucose disaccharide, accumulation provided enhanced resistance to aphids [51, 52]. Stachyose can increase the osmotic pressure in the phloem and interfere with sap acquisition by aphids, which could be used as a defense mechanism in maize plants against aphids [53]. However, some aphids have developed a strategy to overcome this osmotic barrier [54], leading here to the diminution of the power of such defense mechanism.

In addition of JA, SA is involved in plant defense signaling and provides resistance to phloem-feeding insects [4, 8, 55]. Our previous work and the data presented in this work are in agreement that the CLA feeding on Mp708 plants significantly induced the expression of genes involved in SA biosynthesis, however, pharmacological studies confirmed that *mir1*-dependent defense against CLA is disengaged from SA pathway [13]. Similarly, several studies have shown that aphid feeding-induced accumulation of SA and/or the expression of SA-related genes appeared as a generalized plant response to aphids, but was not critical for controlling aphid infestation on host plants [56–59]. Collectively, data presented in this study reiterates that Mp708’s timely transcriptional reprogramming leads to activate a robust defense machinery against CLA invasion.

### Involvement of other defense mechanisms

CLA susceptible Tx601 genotype had a significantly higher content of sesquiterpenoid compounds compared to Mp708 genotype. Terpenoids, sesquiterpenoids, and related VOCs have been demonstrated to be involved in indirect plant defense mechanisms against herbivores or pathogens. Here, sesquiterpene compounds were enriched in the uninfested leaves of the susceptible Tx601 genotype. Our transcriptomic study revealed that several *TPS* genes were downregulated at 24 hpi in both genotypes, except for *TPS5* and *TPS23*. Both *TPS5* and *TPS23* were significantly upregulated in the leaves of the resistant Mp708 genotype prior to aphid infestation. In maize, the induction of *TPS23* was found to be associated with the attraction of natural enemies of herbivores by controlling (E)-β-caryophyllene emissions [60]. However, *TPS23* was significantly downregulated in Mp708 leaves after CLA infestation and not expressed in Tx601 infested conditions (roots and leaves) and in roots of Mp708 infested plants. *TPS2* and *TPS3*, related to linalool synthesis, have been previously identified induced in B73 plants after CLA infestation [61], however, both genes were significantly downregulated in Tx601 plants, suggesting an inactivation of the linalool synthesis pathway after aphid attack. Previously, it was shown that herbivore susceptible horsenettle (*Solanum carolinense*) plants had higher constitutive volatile emissions, but weaker induction of volatiles after insect infestation [62]. Similarly, it is plausible that increased constitutive volatile emissions in Tx601 genotype does not translate to a corresponding increase in CLA feeding-induced volatiles. Although it was shown that the attractiveness of wasp, which parasitizes lepidopteran larvae feeding on maize, exhibited a preference for the sesquiterpene blend that had a mixture of both constitutive and herbivore-induced volatiles [63], it is equally likely that constitutively emitted volatiles may act antagonistically with the herbivore-induced plant volatiles to attenuate the attraction of natural enemies. Alternatively, unlike Mp708 plants that rapidly mounts appropriate defenses (e.g., Mir1-CP, plant defense pathways), CLA susceptible Tx601 plants that fails to induce direct defense mechanisms may emit constitutive volatiles to attract natural enemies. However, in a susceptible host, insects are also able to suppress effective indirect defenses [64]. Collectively, considering that many *TPS* genes were significantly upregulated after CLA infestation in the resistant Mp708 genotype, we hypothesize that Mp708 plants will exhibit stronger induction of CLA feeding-induced volatiles, thereby considerably attracting more predatory insects.

## Conclusions

The susceptible and resistant maize genotypes were altered at the transcriptomic and volatile profile levels before and after aphid infestation. At the transcript level, the resistant Mp708 plants had a more efficient response following CLA herbivory. Genes encoding several proteins required for phytohormone biosynthesis and TFs potentially linked to hormone signaling were upregulated in the resistant Mp708 plants both prior to and after CLA infestation. These data suggest that the resistant maize genotype possessed a finer regulation of the plant hormonal pathways and VOCs, potentially leading to enhanced resistance to CLA.

## Methods

### Plant cultivation and aphid propagation

Corn leaf aphids and maize plants were grown as described previously [13, 18]. Both Mp708 and Tx601 plants for the experiments were used at the V2-V3 developmental stage (~ 2 weeks) [65] and were grown in 3.8 cm × 21.0 cm plastic Cone-tainers (Hummert International, Earth City, MO). At the V2-V3 stage, the second true leaf of maize plants were infested with 10 adult CLA. CLA-infested leaves were clip-caged and tissues were collected 24 hpi. CLA uninfested samples were collected at 0 hpi as control plants. Nine plants were infested with three replicates formed by pooling three samples per replicate. For root collection, plants were carefully removed from the soil and roots samples collected as described previously [13, 14].

### RNA extraction and RNA-seq libraries construction and sequencing

Maize root and leaf tissues (80–100 mg) were ground using 2010 Geno/Grinder® (SPEX SamplePrep, NJ, USA) for 40 s at 1400 strokes min^− 1^ in the presence of liquid nitrogen. Subsequently, total RNA was extracted from the homogenized tissue using Qiagen RNeasy Plant Mini Kit. Nanodrop 2000c Spectrophotometer (Thermo Scientific TM) was used to quantify the extracted total RNA. Then, RNA-seq libraries were constructed on an Illumina HiSeq 2500 (University of Minnesota Genomics Center) using the mRNA-seq standard TruSeq protocol. RNA-seq libraries were sequenced in 50 bp paired-end with 20 million reads on average per library.

### Analysis of RNA-seq libraries

The quality check of the RNA-seq libraries was performed with FASTQC [66] and reads with a Phred score lower than 20 and length below 45 base pairs were removed with Trimmomatic v0.39 [67]. Then, trimmed reads were mapped on the maize reference genome v4 (https://phytozome.jgi.doe.gov/pz/portal.html#!bulk?org=Org_Zmays) with Tophat2 [68] using the following parameters: 1 mismatch (−N 1), 0 splicing mismatch (−m 0), unique mapped reads (−g 1 -M). The output statistics of the trimming and mapping are summarized in the Supplemental Table 6. The transcripts reconstruction was performed with Cufflinks v2.2.1 with the following parameters: quantification against the reference annotation only (−G), multi-read-correct (−u) and frag-bias-correct (−b). The differential expressed gene analysis was performed with Cuffdiff 2.2.1. DEGs were identified with the following parameters: *P* ≤ 5% and false discovery rate |log_2_(Infested/Contol)| ≥ log_2_ [2]. Gene ontology (GO) were analyzed with MaizeMine (http://128.206.234.22:8080/maizemine/begin.do) by using the reference annotation as a template. Hierarchical clustering was performed using the Hierarchical Clustering Explorer 3.5 software (http://www.cs.umd.edu/hcil/hce/hce3.html) with the complete linkage method and the Pearson correlation coefficient. The minimal similarity to establish the clusters was set to 0.834, which is the Pearson correlation significant at the *P*-value threshold of 0.01. PCA analysis has been performed in R with the ggplot package.

### Maize volatile collection and analysis

VOCs that were constitutively emitted from Tx601 and Mp708 plants without CLA infestation were collected using a push-pull system [69]. Eight plants in the V3 stage of each genotype were enclosed in individual glass chambers (20 cm diameter and 30 cm height) resting on teflon guillotine-style bases that excluded contamination of odors from the soil. Air that was purified with activated charcoal was pushed into each chamber at 2 L min^− 1^ and the volatiles emitted from the plants were collected by pulling air from each chamber at the rate of 1 L min^− 1^, over volatile filter traps containing HayeSepQ (Sigma Aldrich, USA). Volatiles were collected for 12 h under constant light of 180 μmol m^− 2^ s^− 1^. Volatile filter traps were eluted with 150 μl of dichloromethane and 5 μL of nonyl acetate (80 ng μL^-1^) as internal standard. One microliter of eluted samples was injected into Agilent 6890 gas chromatograph and 5973 mass spectrometer with a splitless injector held at 250 °C. After sample injection, the column (Rxi®-1 ms, 30 m, 0.25 mm id, 0.25 μm filmthickness; Restek, USA) was maintained at 40 °C for 2 min after which temperature was increased 10 °C per minute until it reached 190 °C and then 12 °C per minute until it reached 280 °C. Identification of target compounds were made by comparison of mass spectra and retention times with published data (NIST14 mass spectral library) with > 90% fidelity by ChemStation (Agilent, USA). All compounds were quantified relative to the nonyl acetate standard.

## Supplementary Information

**Additional file 1: Supplemental Table 1**: DEGs expression level and cluster-geneID link.

**Additional file 2: Supplemental Table 2**: Cluster gene function enrichment.

**Additional file 3: Supplemental Table 3**: TFs expression level.

**Additional file 4: Supplemental Table 4**: DEGs expression level of genes involved in hormone pathways.

**Additional file 5: Supplemental Table 5**: DEGs expression level of TPS genes.

**Additional file 6: Supplemental Table 6**: Trimming and mapping outputs.

**Additional file 7: Supplemental Figure 1**: Principal component analysis (PCA) of the volatile organic compounds (VOCs). Mp708 and Tx601 plants are denoted with blue and turquoise, respectively.

## Data Availability

The raw datasets generated during the sequencing of current study are available as BioProject: PRJNA661336, and is available at the following link: https://dataview.ncbi.nlm.nih.gov/object/PRJNA661336?reviewer=3m1u79lr3svuom44fqnfasv4kn

## Abbreviations

CLA: Corn Leaf Aphid
DEGs: Differentially Expressed Genes
ET: Ethylene
FAW: Fall Armyworm
FC: Fold-Change
FDR: False Discovery Rate
GC-MS: Gas Chromatography-Mass Spectrometry
GO: Gene Ontology
HPI: Hours Post Infestation
JA: Jasmonic acid
LOX: Lipoxygenase
OPDA: 12-oxo-phytodienoic acid
SA: Salicylic acid
TF: Transcription Factor
TPS: Terpene Synthase
VOCs: Volatile Organic Compounds
