# Supplementary material for: Transcriptomic and volatile signatures associated with maize defense against corn leaf aphid
Source: BMC Plant Biol. 2021 Mar 16;21:138. doi: 10.1186/s12870-021-02910-0 (PMC7968207; doi:10.1186/s12870-021-02910-0)
Supplement: Supplementary file 7 — Additional file 7: Supplemental Figure 1: Principal component analysis (PCA) of the volatile organic compounds (VOCs). Mp708 and Tx601 plants are denoted with blue and turquoise, respectively. [file 12870_2021_2910_MOESM7_ESM.pptx]

## Slide 1
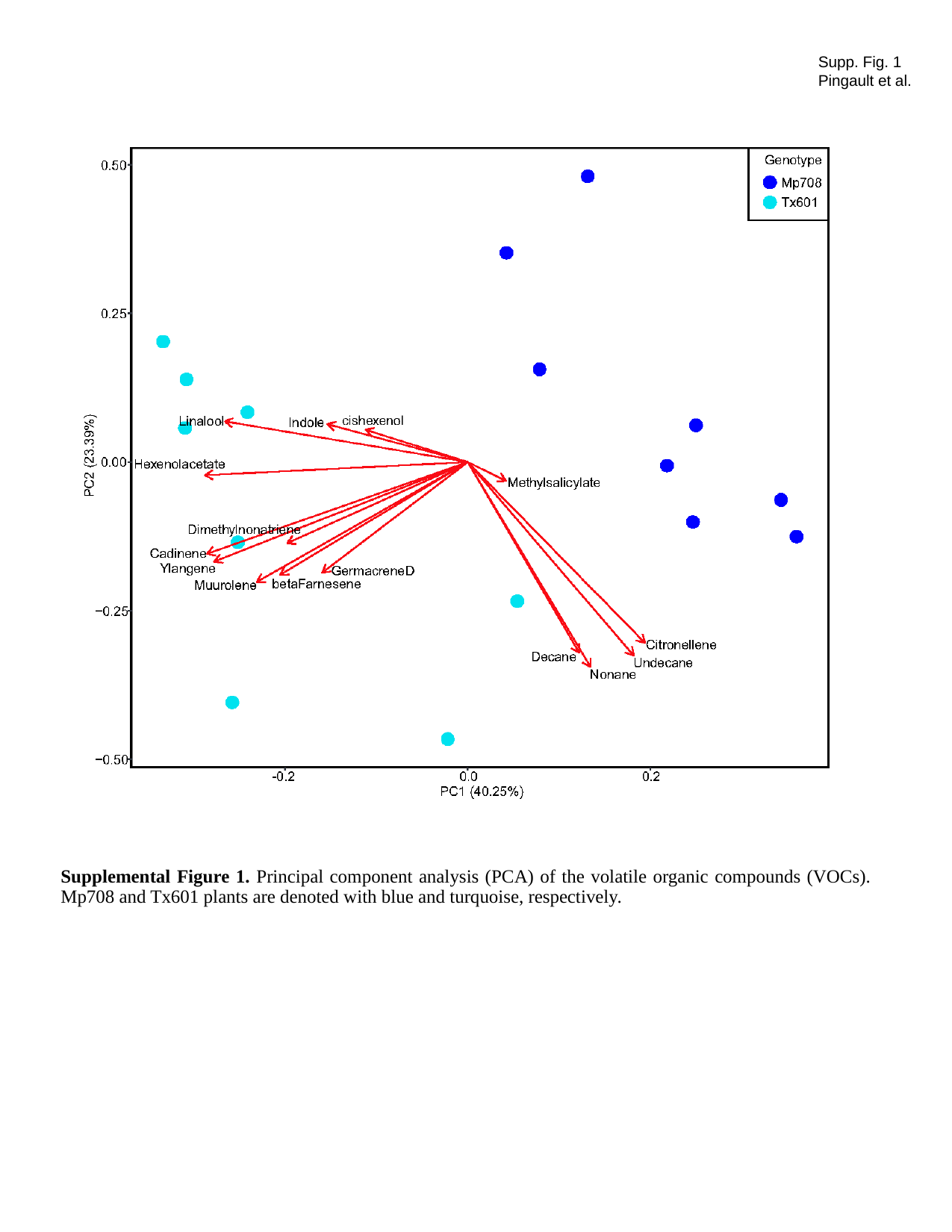

Supp. Fig. 1
Pingault et al.
Supplemental Figure 1. Principal component analysis (PCA) of the volatile organic compounds (VOCs). Mp708 and Tx601 plants are denoted with blue and turquoise, respectively.
